# A Highly Efficient Fluorescent Turn-Off Nanosensor for Quantitative Detection of Teicoplanin Antibiotic from Humans, Food, and Water Based on the Electron Transfer between Imprinted Quantum Dots and the Five-Membered Cyclic Boronate Esters

**DOI:** 10.3390/molecules29174115

**Published:** 2024-08-30

**Authors:** Yansong Zhang, Daojin Li, Xiping Tian

**Affiliations:** 1 School of Food and Drug, Luoyang Normal University, Luoyang 471934, China; 2 Henan Key Laboratory of Fuction-Oriented Porous Materials, College of Chemistry and Chemical Engineering, Luoyang Normal University, Luoyang 471934, China; tianxiping@lynu.edu.cn

**Keywords:** CdTe quantum dot, molecularly imprinted polymer, boronate esters, fluorescent sensor, teicoplanin

## Abstract

Teicoplanin has been banned in the veterinary field due to the drug resistance of antibiotics. However, teicoplanin residue from the antibiotic abuse of humans and animals poses a threat to people’s health. Therefore, it is necessary to develop an efficient way for the highly accurate and reliable detection of teicoplanin from humans, food, and water. In this study, novel imprinted quantum dots of teicoplanin were prepared based on boronate affinity-based precisely controlled surface imprinting. The imprinting factor (IF) for teicoplanin was evaluated and reached a high value of 6.51. The results showed excellent sensitivity and selectivity towards teicoplanin. The relative fluorescence intensity was inversely proportional to the concentration of teicoplanin, in the range of 1.0–17 μM. And its limit of detection (LOD) was obtained as 0.714 μM. The fluorescence quenching process was mainly controlled by a static quenching mechanism via the non-radiative electron-transfer process between QDs and the five-membered cyclic boronate esters. The recoveries for the spiked urine, milk, and water samples ranged from 95.33 to 104.17%, 91.83 to 97.33, and 94.22 to 106.67%, respectively.

## 1. Introduction

**Figure 1:**
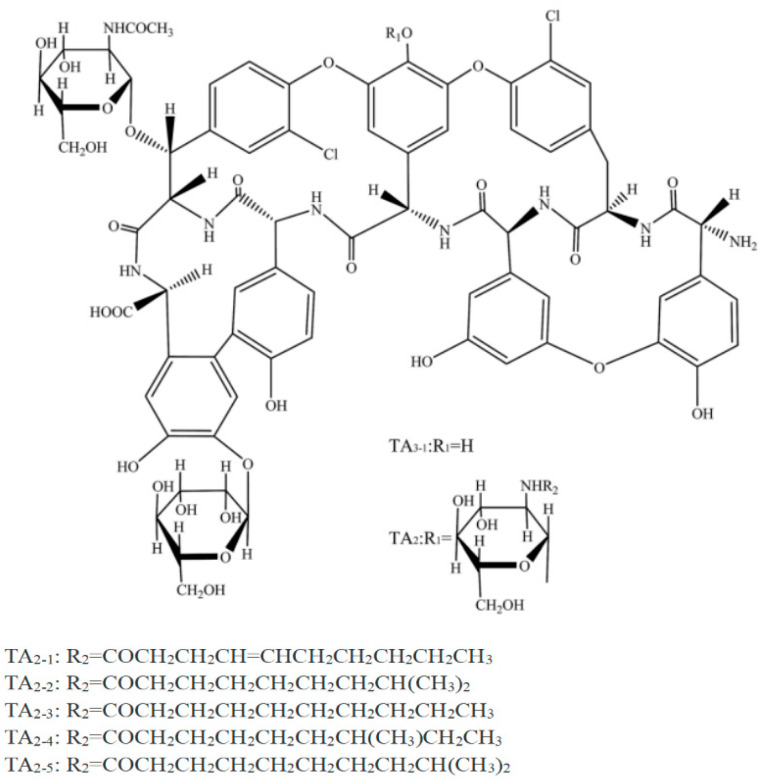
Structure of teicoplanin.

Teicoplanin, also known as Tei, is a glycopeptide antibiotic derived from natural sources. It consists primarily of five compounds (A2-1, A2-2, A2-3, A2-4, and A2-5) with very similar structures that closely resemble each other [1]. A2-2 is the predominant component, constituting over 50% of the A2 series mixture (Figure 1). Despite the widespread use of antibiotics for treating various bacterial infections [2,3], the global community has recognized bacterial drug resistance as a significant public health concern [4,5]. Glycopeptide antibiotics are regarded as the final option for treating severe Gram-positive bacterial infections in clinical settings [6]. Tei, being an important glycopeptide antibiotic, clearly exhibits outstanding antibacterial properties against Gram-positive bacteria. In vivo, Tei exhibits greater antibacterial activity compared to vancomycin. Furthermore, Tei exhibits decreased nephrotoxicity compared to other alternatives and has a prolonged elimination half-life. Considering the issue of antibiotic drug resistance, glycopeptide antibiotics, including Tei, have been prohibited in the veterinary field by the European Union (EU), China, and several other countries. However, the abuse of Tei by humans and edible animals and the resulting Tei residue will pose a threat to people’s health. It is quite important and necessary to develop an efficient way for the highly accurate and reliable detection of Tei from human, animal-derived food, and environmental samples.

Various qualitative–quantitative methods, such as immunoassays, HPLC with UV or electrochemical detection, and HPLC-MS/MS, have been employed for Tei analysis in real samples [7,8,9,10,11,12,13,14]. Although widely used for their high sensitivity and accuracy, traditional analysis methods face challenges, such as expensive instruments, complex sample preparation, and long processing times. In contrast, fluorescence-based chemical sensors offer higher sensitivity, faster response times, simpler operation, and lower costs. Semiconductor quantum dots (SQDs) are commonly used in these sensors due to their high photostability, brightness, and luminescence efficiency [15]. However, an SQDs-based fluorescent sensor for Tei has not yet been developed.

The high specific recognition of the target compound is critical for fluorescence detection. Molecularly imprinted polymers (MIPs) are incorporated into SQDs-based fluorescent sensors [16,17,18,19,20,21,22,23,24,25,26,27] to enhance the selectivity and binding affinities of these fluorescence sensors. However, it is challenging to effectively control the thickness of the imprinting layer using conventional methods, which can impact the efficiency of template removal and the overall imprinting effect. To address this issue, a boronate affinity-based precisely controlled surface imprinting approach was implemented [28,29,30,31,32,33]. This strategy can also be applied to Tei, as the cis-diol of Tei can form a covalent bond with boronic acids through boronate affinity interaction. It is known that boronic acids can undergo reactions with cis-diols at higher pH values and the complex dissociates at low pH values [16,34,35,36,37,38,39]. The imprinting method provides a higher effect and efficiency in template removal compared to the conventional method. This is due to the precise control of the imprinting coating on the surface of QDs, which enhances the specific recognition of Tei.

A boronate affinity-based precisely controlled surface imprinting approach was applied to prepare a molecularly imprinted coating for Tei on the surface of boronic acid-functioned CdTe QDs in this study. The boronate affinity-based precisely controlled surface imprinting approach is more preferable for removing the template compared to other imprinting methods. The other unique strength of the approach is that imprinting the coating can cover excessive binding sites on boronic acid-functionalized CdTe QDs, effectively eliminating non-specific adsorption and offering greater imprinting efficiency. The sensitive and selective determination of Tei from urine, milk, and water samples was demonstrated by the developed fluorescent turn-off nanosensor. Moreover, the imprinting technique is versatile and efficient for all glycopeptide antibiotics.

## 2. Results and Discussion

### 2.1. Characterization of QDs@APBA and QDs@APBA@MIPs

**Figure 2:**
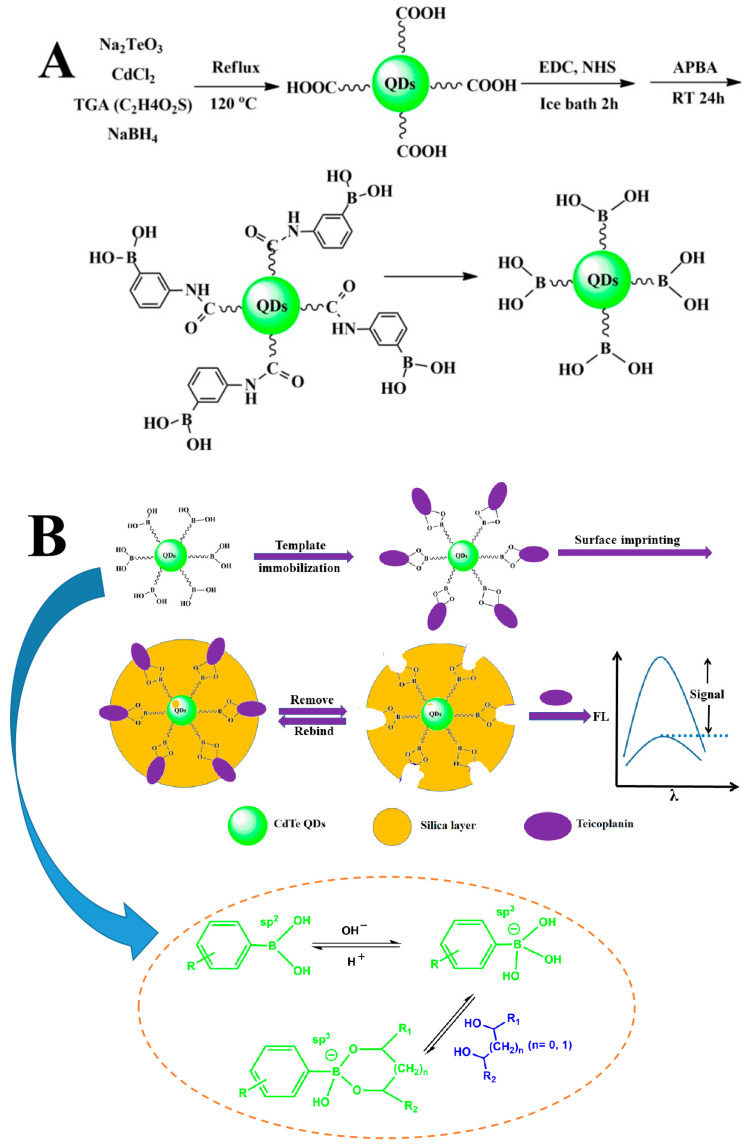
Synthesis routes of APBA-functionalized CdTe QDs (**A**) and the formation mechanism of Tei-imprinted magnetic nanoparticles by boronate affinity-based precisely controlled surface imprinting (**B**).

**Figure 3:**
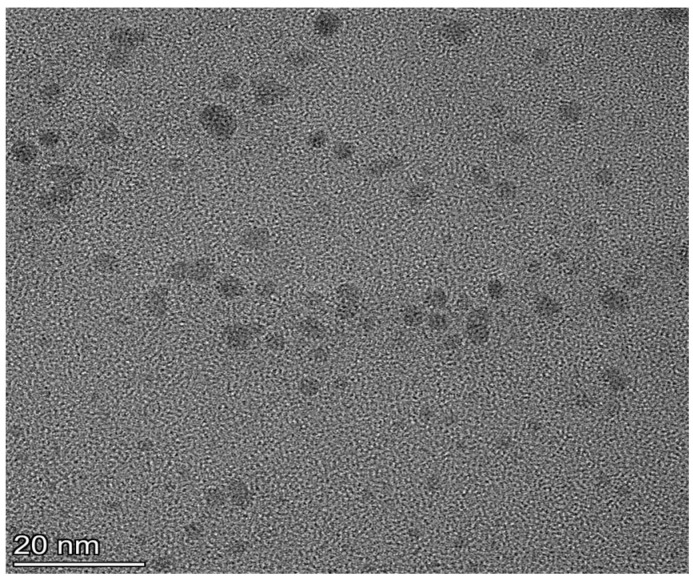
Transmission electron microscopy characterization (TEM) of imprinted APBA-functionalized CdTe QDs.

The imprinting coating process is illustrated in Figure 2. First, the Tei template is attached to boronic acid-functionalized CdTe QDs through boronate affinity interaction. Then, a suitable thickness of the imprinting layer is coated onto the substrate surface via self-polymerization of TEOS. Finally, the template is eluted using an acidic solution with SDS to create 3D cavities with boronic acid groups that complement the molecular size and shape of the Tei template. This results in obtaining imprinted APBA-functionalized CdTe QDs (QDs@APBA@MIPs) with fluorescence. After the specific binding of Tei to the 3D cavities with APBA, the fluorescence intensity is quenched by Tei to some extent. The morphology of QDs@APBA@MIPs was investigated using transmission electron microscopy (TEM). As shown in Figure 3, the QDs@APBA@MIPs showed a high level of uniformity in morphology and an exceptional ability to disperse in water. The particle size measured is approximately 6–8 nm, showcasing its minuscule and intricate nature.

In addition, QDs@APBA were prepared by the covalent coupling of an APBA molecule with bare CdTe QDs via an amidation reaction between carboxyl and amino. The investigation of the fluorescence intensity changes of bare CdTe QDs and QDs@APBA at different concentrations of Tei was conducted as a means to prove the successful post-modification reactions. Figure S1A shows that the fluorescence quenching in QDs@APBA was greater than in bare CdTe QDs, indicating successful modification with APBA. Additionally, the selectivity for cis-diols was confirmed by evaluating changes in fluorescence intensity for Tei, Rut, and Bai (cis-diols) compared to DA, Gen, and Iso (non-cis-diols), as shown in Figure S1B. This confirms the successful conjugation of boronic acid onto the carboxyl-capped CdTe QDs.

**Figure 4:**
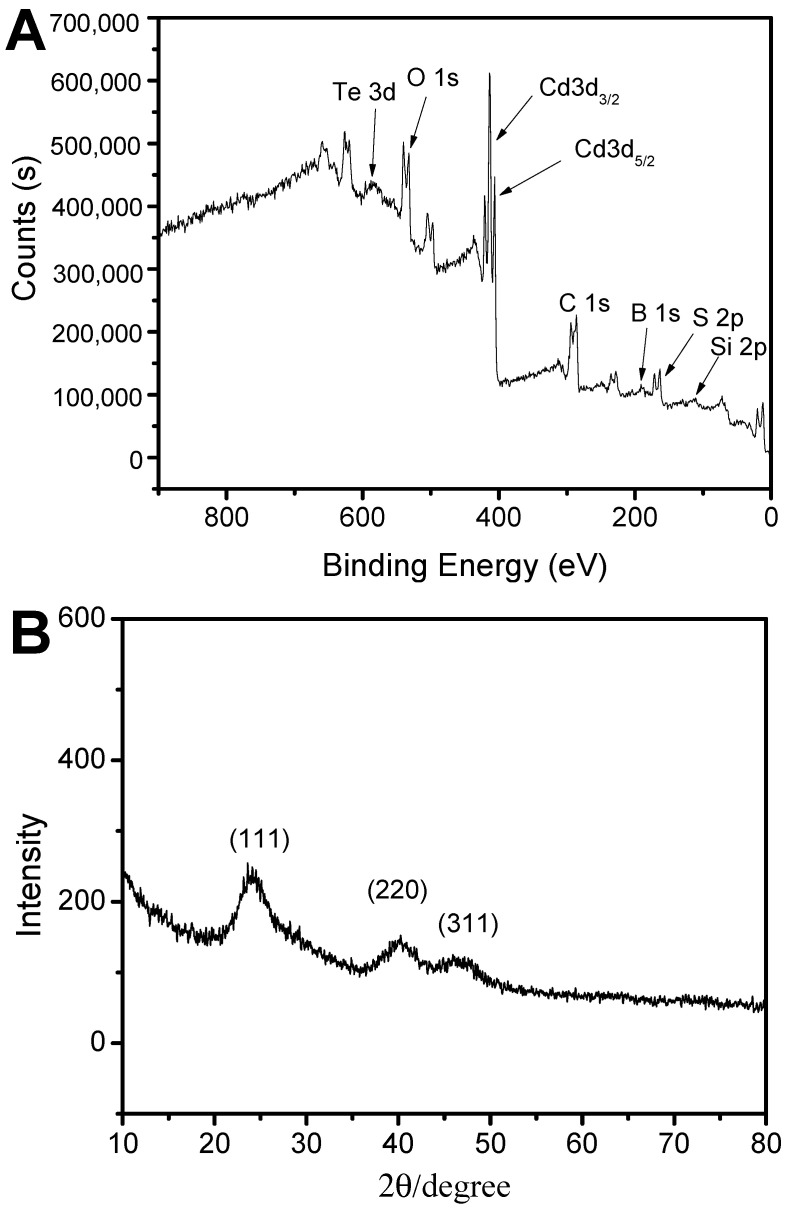
XPS (**A**) and XRD (**B**) spectra of QDs@APBA@MIPs.

The X-ray photoelectron survey spectra (XPS) were used to examine the surface composition of the QDs@APBA@MIPs. Figure 4A demonstrates the use of XPS to analyze the surface composition of the QDs@APBA@MIPs. The peaks observed at 407.1 eV and 413.1 eV in the XPS spectrum of Figure 4A are assigned to Cd3d5/2 and Cd3d3/2, respectively. Similarly, the peak at 585 eV is assigned to Te3d. Furthermore, the XPS analysis revealed peaks at 164.1 eV for S2p, 286.1 eV for C1s, 532.1 eV for O1s, 190.1 eV for B1s, and 104.1 eV for Si2p. The signals of Cd3d5/2, Cd3d3/2, Te3d5/2, Te3d3/2, and S2p confirm the presence of CdTe QDs in the nanomaterials. The peak at 190.1 eV is attributed to B atoms from boronic acid, demonstrating the successful post-modification of boronic acid onto the CdTe QDs. The Si2p peak confirmed that the silica imprinting layer was prepared successfully. The successful preparation of QDs@APBA@MIPs is clearly indicated by these results. In addition, the XRD pattern for QDs@APBA@MIPs (Figure 4B) showed characteristic peaks that verified the nanocrystalline structure of the CdTe QDs. Three main peaks were observed, located at the 2θ values of about 24.6°, 41.1°, and 48.3° which were related to the (111), (220), and (311) planes, respectively. The results align with the findings from previous research [40], which showed that the structure of the CdTe QDs carrier remained unchanged after post-modification and imprinting.

In order to further verify the boronic acid functionalization of bare CdTe QDs and subsequent molecular imprinting, the fluorescence spectra of bare CdTe QDs, QDs@APBA, and QDs@APBA@MIPs were investigated. As shown in Figure S2, QDs@APBA was weaker compared to bare CdTe QDs. The fluorescence signal of QDs@APBA@MIPs was also observed to be weaker than that of QDs@APBA. This phenomenon may be attributed to energy transfer from CdTe QDs to APBA, as well as the imprinting coating leading to the appearance of new defects on the surfaces of QDs. These results provide evidence for the successful modification reactions and imprinting coating.

### 2.2. Optimization of the Polymerization Time

The structure of nanoscale imprinted 3D cavities in MIPs presents a high level of complementarity to the imprinting templates, as we are aware. Thus, the surface-imprinted CdTe QDs towards the template possessed good specific affinity due to the well-fabricated imprinting cavities from the imprinting coating-formed silica. Furthermore, boronic acids have the capability to form stable five- or six-membered cyclic boronate esters through covalent binding with cis-diol groups, like Tei, at relatively high pH levels. These esters can be reversibly dissociated under acidic conditions. Thus, a combination of imprinted cavities and boronic acids can lead to good binding properties.

**Figure 5:**
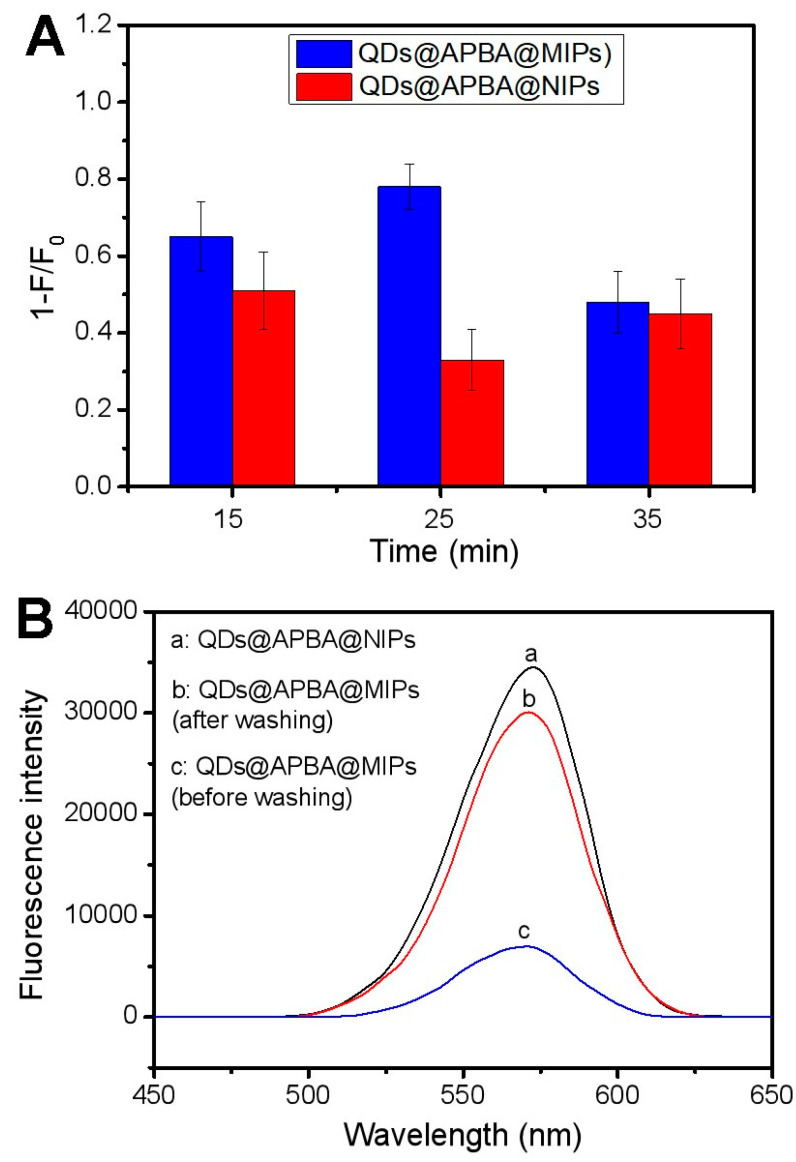
(**A**) The dependence of imprinting effect on polymerization time for preparing imprinted APBA-functionalized CdTe QDs and non-imprinted APBA-functionalized CdTe QDs, [Tei] = 1.7 × 10^−5^ M; (**B**) fluorescence emission spectra of QDs@APBA@NIPs (a), QDs@APBA@MIPs (after washing, b), QDs@APBA@MIPs (before washing, c).

The thickness of the imprinting coating is key, relating to the binding properties of the imprinted QDs, because it can influence the size and shape of the imprinted 3D cavities for Tei. In general, the imprinting coating should be thinner than the template’s molecular size. In this study, a silica layer formed by the polycondensation of TEOS was chosen as the imprinting coating for two reasons. First, it is relatively hydrophilic and can reduce non-specific adsorption. Second, it causes less fluorescence quenching of boronic acid-functionalized CdTe QDs compared to organic polymer layers such as poly(dopamine), poly(2-anilinoethanol), or poly(aniline). The thickness of the imprinting coating is directly proportional to the polycondensation time at 10–60 min [41]. Therefore, we selected 15, 25, and 35 min as assessment objects during the imprinting process based on the size of the Tei molecule. The imprinting effect was evaluated by comparing the fluorescence quenching caused by Tei in QDs@APBA@MIPs with that in non-imprinted APBA-functionalized CdTe QDs (QDs@APBA@NIPs). As shown in Figure 5A, the best imprinting effect was observed with a polymerization time of 25 min. These imprinting conditions were subsequently utilized in the other experiments to obtain the most efficient QDs@APBA@MIPs.

After the template molecules were removed from the composites by decomposing the boronic acid esters bonds, the specific cavity with binding sites was formed, and the QDs@APBA@MIPs were obtained. The fluorescence intensity of the QDs@APBA@MIPs was approximately 20.19% of the QDs@APBA@NIPs before the removal of the template molecules. However, after removal, the fluorescence intensity increased to 87.03% (Figure 5B). This result indicated that the template molecule Tei was nearly removed thoroughly from the composites, which exhibited high imprinting efficiency caused by boronate affinity-based precisely controlled surface imprinting.

### 2.3. Static Adsorption Test

**Figure 6:**
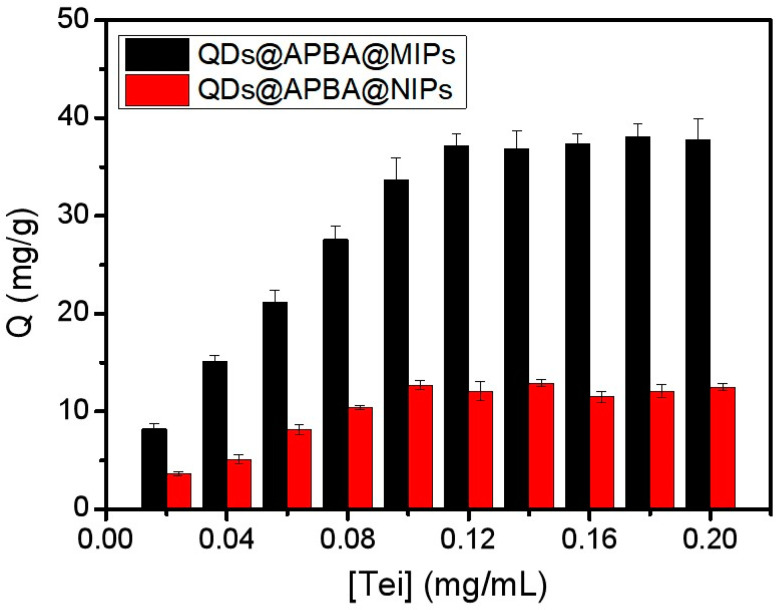
Comparison of adsorption performance of QDs@APBA@MIPs and QDs@APBA@NIPs.

The adsorption performance of MIPs is an important indicator, which greatly affects MIPs-based fluorescent sensors. As depicted in Figure 6, the adsorption capacities of QDs@APBA@MIPs and QDs@APBA@NIPs for Tei increased as the Tei concentration increased. However, the adsorption capacity of QDs@APBA@MIPs was noticeably higher than that of QDs@APBA@NIPs. This is attributed to the presence of three-dimensional imprinted cavities in QDs@APBA@MIPs, which were formed due to molecular imprinting. The adsorption isotherms demonstrated that QDs@APBA@MIPs had a maximum adsorption capacity of 38.1 mg/g.

### 2.4. Fluorescence Quenching of QDs@APBA@MIPs by Tei

**Figure 7:**
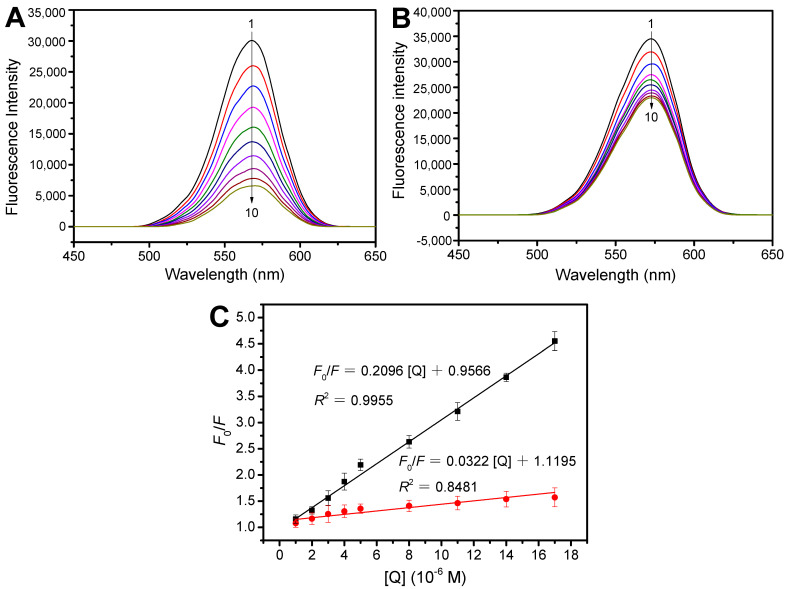
Fluorescence emission spectra of the QDs@APBA@MIPs (**A**), the QDs@APBA@NIPs (**B**) with various concentrations of Tei and Stern–Volmer plots (**C**) for the QDs@APBA@MIPs, and the QDs@APBA@NIPs with the target Tei. The concentrations were 0.1 mg/mL; [Tei] = 0, 1, 2, 3, 4, 5, 8, 11, 14, 17 (1 × 10^−6^ mol/L) (1–10).

To explore their potential application in quantitatively determining Tei in real samples, the fluorescence intensities of prepared QDs@APBA@MIPs were studied. Both imprinted and non-imprinted CdTe QDs showed some degree of fluorescence quenching by Tei. However, the extent of fluorescence quenching caused by Tei was much higher for QDs@APBA@MIPs than for QDs@APBA@NIPs (Figure 7). This suggests that QDs@APBA@MIPs have a higher binding capacity for Tei, indicating successful preparation of the imprinted CdTe QDs.

The following Stern–Volmer equation was analyzed to confirm the successful imprinting and further understand the quenching mechanism caused by Tei [42,43,44]:

(1) $$\frac{F_{0}}{F}=1+k_{q}\tau_{0}[Q]=1+K_{SV}[Q]$$

In the absence and presence of template Tei, *F*_0_ and *F,* respectively, indicate the fluorescence intensity of QDs@APBA@MIPs. [*Q*] represents the concentration of quencher Tei. *K*_SV_ refers to the Stern–Volmer dynamic quenching constant, and *k_q_* represents the kinetic constant of the quenching process. *τ*_0_ denotes the fluorescence lifetime, when there is no quencher Tei. The linear regression curves for QDs@APBA@MIPs and QDs@APBA@NIPs are shown in Figure 7C, with the resulting regression equations evaluated as *F*_0_/*F* = 0.2096 [*Q*] + 0.9566 and *F*_0_/*F* = 0.0322 [*Q*] + 1.1195, respectively. Clearly, the *K_SV_* values for MIPs and NIPs were determined to be 2.096 × 10^5^ M^−1^, and 3.220 × 10^4^ M^−1^, respectively. The imprinting factor (IF), also known as *K_SV_*,_MIP_/*K_SV_*,_NIP_, was utilized to evaluate the imprinting effect of the fluorescence sensors obtained. A computed IF value of 6.51 demonstrates the effectiveness of the imprinting process. The findings suggest that the nanosensor based on MIPs can greatly enhance the selectivity and efficiency of quenching for Tei. Furthermore, the QDs@APBA@MIPs demonstrated a linear decrease in quenching intensity when exposed to Tei concentrations ranging from 1.0 to 17 μM. The sensitivity of the proposed sensing platform was evaluated in terms of the limit of detection (LOD). The LOD of the Tei was calculated to be 7.14 × 10^−7^ M, according to the formula of LOD = 3/N. The mean standard deviation of the intercept was calculated as 0.05, and N is the slope of the calibration curve.

**Figure 8:**
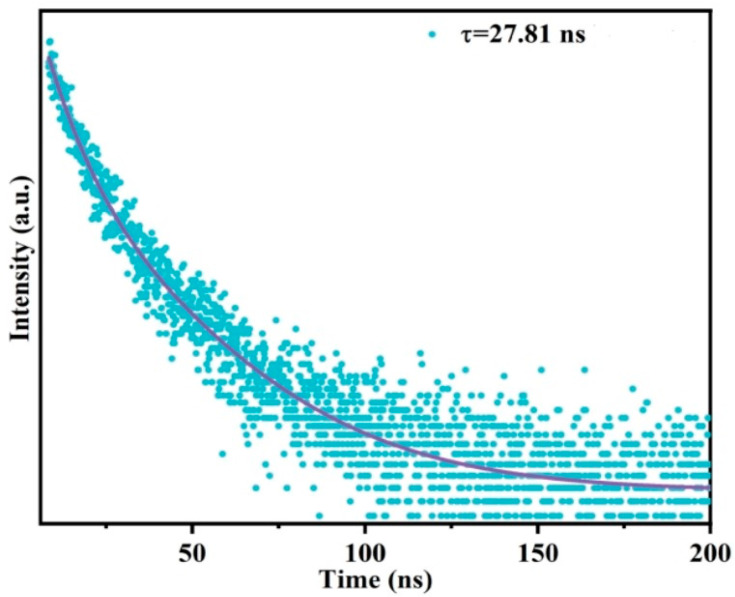
Fluorescence decay curves for QDs@APBA@MIPs.

To elucidate and confirm the quenching mechanism of the reaction between the QDs@APBA@MIPs and Tei, the fluorescence lifetime of the QDs@APBA@MIPs was first analyzed. The linear Stern–Volmer plot (Figure 7C) may suggest that either a static or dynamic process occurs. The fluorescence lifetime measurement can differentiate the two quenching processes. Figure 8 shows the fluorescence decay curve of QDs@APBA@MIPs, with a *τ*_0_ of 27.81 ns. Thus, the calculated *k_q_* value is 7.54 × 10^12^ L mol^−1^ s^−1^, which is 3 orders of magnitude greater than the *k_q_* value in water at room temperature (7.4 × 10^9^ L mol^−1^ s^−1^) [45]. The result indicated a static quenching mechanism, not a dynamic quenching process. The primary factor influencing dynamic quenching is diffusion in most cases. As the diffusion coefficient becomes larger with the higher temperature, the dynamic quenching constant also would increase accordingly. In contrast, static quenching would lead to a decrease in the quenching rate constant with an increase in the temperature. The results shown in Figure S3 indicate that *K*_SV_ is negatively correlated with temperature, suggesting that Tei-induced quenching is not due to dynamic collision but rather the formation of a complex.

### 2.5. The Operative Mechanism of Static Fluorescence Quenching

As noted previously, the primary control of the fluorescence quenching process is through a static quenching mechanism. In the static quenching process, either the energy transfer or the electron transfer occurs. To determine the operative mechanism of static fluorescence quenching, we measured the absorption spectrum of Tei and the emission spectrum of the QDs@APBA@MIPs, as shown in Figure S4. It is evident that there was no spectral overlap observed between the UV-Vis absorption spectrum of Tei and the fluorescence emission spectrum of QDs@APBA@MIPs. This outcome failed to satisfy the requirements of Förster resonance energy transfer (FRET). Hence, the energy-transfer mechanism can be eliminated as a cause for fluorescence quenching. It implied that the electron transfer from the QDs@APBA@MIPs to the Tei could be the main optosensing turn-off mechanism. In this scenario, there might have been a non-radiative electron-transfer process between the QDs and the cyclic boronate esters [46,47]. This non-radiative recombination has been associated with the presence of trap states, specifically five-membered cyclic boronate esters [48], resulting in the suppression of QDs fluorescence. Therefore, this sensing platform can also be used for the quantitative analysis of Tei.

### 2.6. Effect of Binding pH on the Imprinting Effect

The charges and structures of the boronic acid ligand, Tei, and silica layer are influenced by the surrounding pH. Therefore, pH plays a crucial role in affecting the interaction between Tei and imprinted QDs. It is essential to investigate the impact of pH on the binding of Tei to QDs@APBA@MIPs. The IF value was applied to assess the target binding properties of the imprinted cavities.

As illustrated in Figure S5, the IF value increased with the pH from 4.0 to 7.0 but decreased after the pH exceeded 7.0. The optimal binding pH was found to be 7.0, with an IF value of 6.51. These results can be explained by two factors. (1) According to the principle of boronate affinity, the binding forces of boronic acid with cis-diols increase as the pH increases. (2) The charges of Tei are affected by pH, as polyphenolic hydroxyl is sensitive to pH. Different pH values result in different ionization states and, under optimal binding conditions, at a negative charge for Tei and boronic acids. In conclusion, QDs@APBA@MIPs exhibit electrostatic repulsion towards Tei to some extent, as the binding forces decrease with an increasing pH value. Furthermore, the silica layer that has been prepared can undergo ionization at higher pH levels, resulting in an impact on the interaction of Tei and QDs@APBA@MIPs. Through the optimization experiment, the optimal binding pH was confirmed to be 7.0, which was used for further experiments.

### 2.7. Binding Specificity of QDs@APBA@MIPs

**Figure 9:**
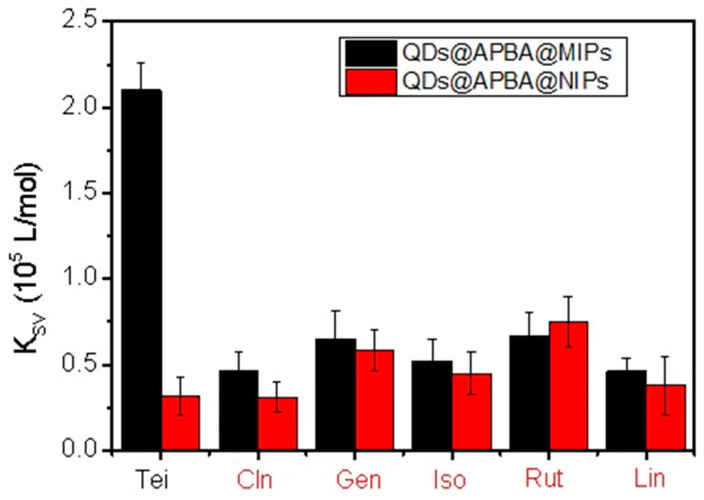
Selectivity of QDs@APBA@MIPs toward Tei using boronate affinity-based precisely controlled surface imprinting.

To evaluate the specificity of QDs@APBA@MIPs for Tei, Stern–Volmer quenching constants for Tei and competitive compounds (Cln, Gen, Iso, Rut, and Lin) were compared in Figure 9. The results clearly showed that QDs@APBA@MIPs demonstrated high specificity of imprinted sensors for Tei.

### 2.8. Reproducibility and Chemical Stability

**Figure 10:**
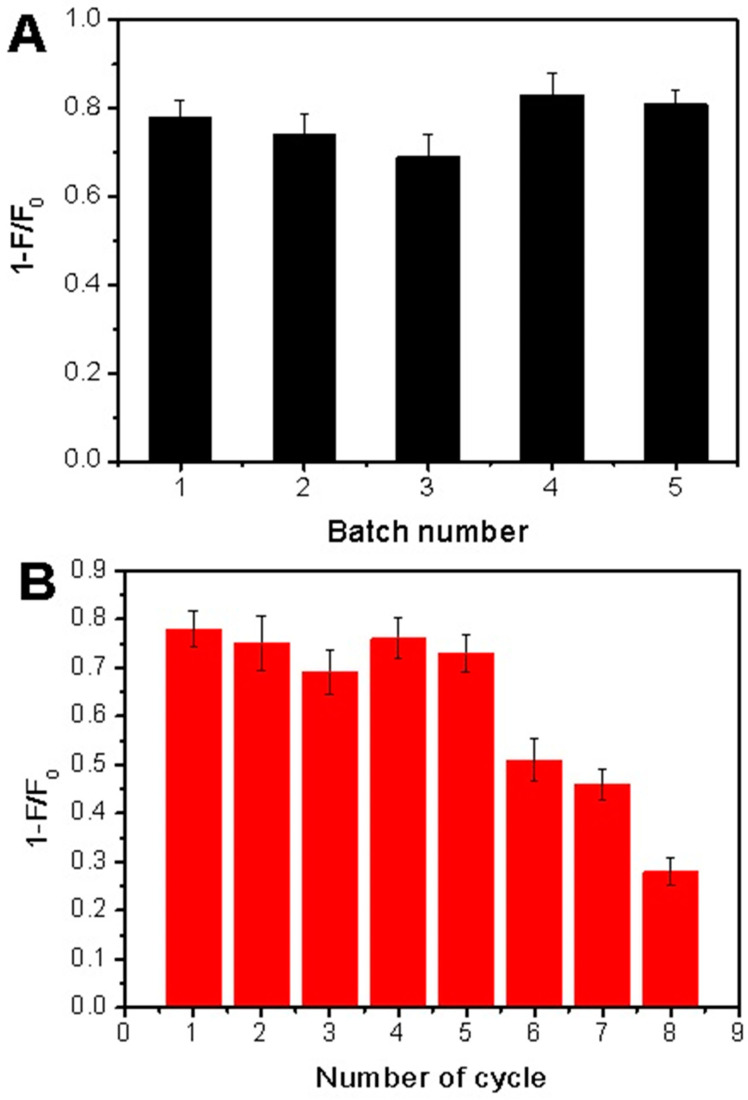
Reproducibility (**A**) and chemical stability of QDs@APBA@MIPs (**B**).

The reproducibility of the Tei-imprinted CdTe QDs as a performing material is vital. Thus, reproducibility was evaluated through the reproducibility experiment. It involved five different batches of QDs@APBA@MIPs prepared at different times, and the measurements were replicated three times in parallel for every batch of samples. As depicted in Figure 10A, each individually prepared QDs@APBA@MIPs exhibited similar fluorescence quenching extent for Tei (1 − *F*_0_/*F*: 0.78, 0.74, 0.69, 0.83 and 0.81, respectively). The outcome demonstrated satisfactory reproducibility of Tei-imprinted APBA-functionalized CdTe QDs.

The MIP-based fluorescent sensor must possess chemical stability as a crucial characteristic. QDs@APBA@MIPs can be reused after removing template Tei with HAc (pH 2.7) due to boronate affinity interactions. The silica materials are very stable at the pH range of 2.0 to 8.0, which favors removing–rebinding templates. To investigate the chemical stability of the surface-imprinted silica layer, the same QDs@APBA@MIPs were subjected to eight cycles of removal and rebinding. As shown in Figure 10B, the developed sensor nearly retained its fluorescence quenching extent for Tei during five removing–rebinding recycles. The fluorescence quenching extent showed a noticeable decrease after more than five repetitions. The possible explanation is that certain specific cavities were obstructed during the binding process or damaged after being rewashed. The findings show that the surface-imprinted silica layer exhibited excellent stability throughout five cycles of removing and rebinding in the fluorescence sensing process.

### 2.9. Determination of Tei in Real Samples

For evaluating the performance of the prepared nanosensor for Tei in real samples, the quantitative analysis of Tei from urine, milk, and water samples by QDs@APBA@MIPs exaction was performed. It can be observed from Table 1 that Tei was not detected in the three samples. In addition, we conducted recovery tests on urine, milk, and water samples. The spiking concentration of Tei was set at three levels, with 3, 6, and 9 μM for each of the above samples. The results achieved were satisfactory, with Tei recovery rates ranging from 95.33 to 104.17% for urine samples, 91.83 to 97.33% for milk samples, and 94.22 to 106.67% for water samples. In addition, the RSD for the urine, milk, and water samples ranged from 3.64 to 5.12, 3.98 to 4.89, and 2.87 to 3.78%. To summarize, the developed QDs@APBA@MIPs have proven to be effective for the rapid and efficient detection of Tei in a real sample analysis.

## 3. Materials and Methods

### 3.1. Reagents and Materials

Na_2_TeO_3_, 1-(3-(dimethylamino)propyl)-3-ethylcarbodiimide hydrochloride (EDC), thioglycolic acid (TGA), N-hydroxysuccinimide (NHS), NaBH_4_, ammonia−water, anhydrous ethanol, teicoplanin (Tei), rutin (Rut), quercetin (Qu), baicalein (Bai), genistein (Gen), isorhamnetin (Iso), CdCl_2_·2.5H_2_O, Tetraethyl orthosilicate (TEOS), and 3-aminophenylboronic acid (APBA) were purchased from Energy Chemical (Shanghai, China).

### 3.2. Instruments

The Jem-2100F system (Jeol, Tokyo, Japan) was employed for TEM characterization. Fluorescence signals from all samples were measured utilizing an F-4500 spectrofluorophotometer (Hitachi, Tokyo, Japan) equipped with 1.0 cm quartz cells. The excitation wavelength was set at 330 nm, with the spectral slit width configured to 2.5 nm. The FLS1000 Edinburgh fluorescence spectrometer is used for fluorescence lifetime measurement. The X-ray photoelectron spectroscopy (XPS) was conducted using an ESCALAB 250Xi X-ray photoelectron spectrometer (Thermo, Waltham, MA, USA) equipped with Al Kα radiation (hv = 1486.6 eV). The instrument was meticulously calibrated against the C1s band at 284.8 eV. Additionally, powder X-ray diffraction (XRD) analysis was carried out on a Bruker D8 Advance diffractometer utilizing Cu Kα radiation (Bruker, Karlsruhe, Germany), and the scanning angle ranged from 10° to 80° of 2θ.

### 3.3. Preparation of APBA-Functionalized CdTe QDs (QDs@APBA)

APBA-functionalized CdTe QDs were prepared in two steps. This involved the synthesis of carboxyl-functionalized CdTe QDs, followed by the boronic acid functionalization of CdTe QDs (Figure 2A). Other researchers have reported on the preparation of bare CdTe quantum dots [41,49]. Simply, CdCl_2_·2.5H_2_O of 182.68 mg was first dissolved in water of 200 mL, followed by the addition of 72 μL TGA, and the pH solution was adjusted to 10.8 using a NaOH solution. After being shaken for several minutes, a mixture solution was combined with 200 mL of water and 39.64 mg of Na_2_TeO_3_. The pH of the solution mentioned above was further modified to a range of 10.5 to 11.0 within a span of 10 min. Afterward, 320 mg of NaBH_4_ were added to the solution and ultrasonicated for 5 min. The resulting solution was then refluxed in an oil bath at 120 °C for 50 min. Excessive absolute ethanol (4 times the volume of the QDs solution) was added, and the mixture was kept for about 3 h before centrifugation. After centrifugation, the QDs solution was washed with ethanol three times and dried at 50 °C in a vacuum oven as the final step.

The boronic acid functionalization of CdTe QDs was conducted following a previous study [29], as shown in Figure 2A. Carboxyl-functionalized CdTe QDs (60 mg) were dispersed in 10 mM phosphate buffer (60 mL, pH 8.0) and sonicated for 3 min. Then, NHS (42 mg) and EDC (82 mg) were added to activate the carboxyl group. After stirring for 2 h, APBA (82 mg) was introduced and shaken for another 2 h in an ice bath. The solution was left at room temperature for 24 h, followed by centrifugation at 9000 rpm for 8 min to precipitate APBA-functionalized CdTe QDs. The resulting QDs@APBA were washed three times with ethanol and dried in a vacuum oven at 50 °C.

### 3.4. Preparation of QDs@APBA@MIPs

QDs@APBA@MIPs were prepared based on the boronate affinity-based precisely controlled surface imprinting through the following synthetic route that included three steps (Figure 2B). First, the Tei template was immobilized onto the surface of CdTe QDs by dispersing it in 25 mL sodium phosphate buffer (pH 7.4) and shaking for 40 min. Ethanol was then added to generate a flocculent precipitate, which was centrifuged at 8000 rpm for 6 min. After that, the APBA-functionalized CdTe QDs were obtained. Second, in order to imprint Tei, the above-mentioned QDs were mixed with ethanol (30 mL) and ammonium hydroxide (0.45 mL) to form a suspension. After that, 7.5 mL TEOS solution of ethanol (10 mM) was added and slightly shaken for a while. The hydrolytic time of TEOS was set at 10, 15, and 20 min to optimize the thickness of the imprinting layer. The CdTe QDs obtained after different hydrolysis times were collected, centrifuged, and washed three times with ethanol. Next, a solution of acetic acid (100 mM) with SDS was used to completely remove Tei from the imprinted QDs until no absorbance was detected, followed by centrifugation. The QDs@APBA@NIPs were used as a blank control. The preparation procedure for QDs@APBA@MIPs was identical to that of QDs@APBA@MIPs, except for omitting the Tei immobilization step. The thickness of the silica imprinting coating on the non-imprinted QDs was the same as that on the imprinted QDs.

### 3.5. Adsorption Performance Test of QDs@APBA@MIPs

Five mL of Tei solution with different concentrations were each mixed with 5 mg of either QDs@APBA@MIPs or QDs@APBA@NIPs. Next, the mixture mentioned above was subjected to 10 min of ultrasonic treatment, followed by shaking at room temperature for 2 h. The concentration of Tei in the supernatant was measured by UV-Vis. The adsorption capacity (Q, mg/g) was calculated using the formula Q = (C_0_ − C)V/m, where C_0_ (mg/mL) and C (mg/mL) represent the initial and final Tei concentrations after adsorption by QDs@APBA@MIPs and QDs@APBA@NIPs, respectively. V is the volume of solution added, and m is the mass of QDs@APBA@MIPs and QDs@APBA@NIPs.

### 3.6. Specificity of the QDs@APBA@MIPs for Tei

The investigation on the specificity of QDs@APBA@MIPs for Tei included research targets such as Tei, Rut, Bai, Qu, Gen, and Iso. Equivalent amounts of QDs@APBA@MIPs and QDs@APBA@NIPs were dispersed in 10 mM phosphate buffer (pH 7.0) and titrated by continuously adding stock solution of each target compound, respectively. Each stock solution had a concentration of 1 mM. After being mixed for 3 min each time, each obtained sample was used for fluorescence measurement.

### 3.7. Application to Real Sample Analysis

The urine sample was obtained from a healthy participant and stored at −20 °C. The item was allowed to thaw at room temperature prior to use. The urine sample, measuring 50 mL, underwent centrifugation for 8 min to eliminate the precipitates. Then, the pH of the obtained supernatant was adjusted to 7.0 using 10 mM phosphate buffer and used as a working sample for analysis.

Milk (50 mL) from local markets in Luoyang was placed in a centrifugal tube, and the pH was adjusted to 7.0 using a 10 mM phosphate buffer. After 10 min of ultrasound treatment, the milk solution became uniform.

5 mg QDs@APBA@MIPs was dispersed in 10 mL of the above obtained urine solution, milk solution, or water, respectively. Different amounts of Tei were added into urine, milk, or water to obtain sample solutions containing Tei at concentrations of 3, 6, and 9 mol/L. The equivalent QDs@APBA@MIPs were then added to a spiked 10 mL solution of urine, milk, or water and rotated for 20 min to induce fluorescence quenching in order to evaluate the recoveries of Tei in the respective solutions.

## 4. Conclusions

The study prepared Tei-imprinted APBA-functionalized quantum dots using boronate affinity-based surface imprinting for precise control. The IF value for Tei was 6.51 under the optimum conditions, implying efficient formation of specific binding sites in Tei-imprinted boronate affinity materials. This made it suitable for quantitatively analyzing Tei. In this sensor, boronate affinity-based MIPs served as recognition elements, while CdTe QDs were used for signal transduction. The concentration of Tei in the range of 1.0–17 μM was found to be inversely proportional to the fluorescence intensity, with a detection limit of 0.714 μM. The developed technique can be applied to determine Tei in real samples. The results showed that the recoveries of Tei for spiked urine, milk, and water samples ranged from 95.33% to 104.17%, 91.83% to 97.33%, and 94.22% to 106.67%, respectively. This method is shown to be effective and provides a new way to develop a sensitive, selective, and rapid determination of Tei.

## Figures and Tables

**Table 1:** Determination of Tei in urine, milk, and water (n = 3).

Samples	Added Quantity (µM)	Detected Quantity ^a^ (µM)	Recoveries (%, *n* = 3)	RSD (%)
Urine	0	0	----	----
	3.00	2.86	95.33	5.12
	6.00	6.25	104.17	3.64
	9.00	8.72	96.89	4.21
Milk	0	0	----	----
	3.00	2.92	97.33	3.98
	6.00	5.51	91.83	4.89
	9.00	8.56	95.11	4.12
Water	0	0	----	----
	3.00	3.20	106.67	2.87
	6.00	5.89	98.17	3.46
	9.00	8.48	94.22	3.78

^a^ Each experiment is repeated three times.

## Data Availability

Data are contained within the article and the Supplementary Materials.

## Supplementary Materials

The following supporting information can be downloaded at https://www.mdpi.com/article/10.3390/molecules29174115/s1: Figure S1: Fluorescence intensity change of CdTe QDs and QDs@APBA with various amounts of Tei (A) and binding behaviors of cis-diol-containing compounds (Tei, Rut, and Bai) and non-cis-diol-containing compounds (DA, Gen and Iso) on the QDs@APBA (B). [Tei] = [Rut] = [Bai] = [DA] = [Gen] = [Iso] = 1.7 × 10^−5^ M; Figure S2: Fluorescence emission spectra of bare CdTe QDs (a), QDs@APBA (b) and QDs@APBA@MIPs (c); Figure S3: Stern–Volmer plots for fluorescence quenching of QDs@APBA@MIPs towards Tei at different temperatures in pH 7.0; Figure S4 The UV–Vis absorption of Tei (1) and fluorescence emission of QDs@APBA@MIPs (2); Figure S5: Effect of pH on imprinting factor (IF) of QDs@APBA@MIPs.
