# Supplementary material for: A Highly Efficient Fluorescent Turn-Off Nanosensor for Quantitative Detection of Teicoplanin Antibiotic from Humans, Food, and Water Based on the Electron Transfer between Imprinted Quantum Dots and the Five-Membered Cyclic Boronate Esters
Source: Molecules. 2024 Aug 30;29(17):4115. doi: 10.3390/molecules29174115 (PMC11397723; doi:10.3390/molecules29174115)
Supplement: Supplementary file 1 [file molecules-29-04115-s001.zip › Supporting Information.pdf]

Supporting Information

A highly efficient fluorescent turn-off nanosensor for quantitative detection of teicoplanin antibiotic from human, food and water based on the electron transfer between imprinted quantum dots and the five-membered cyclic boronate esters

Yansong Zhang*[a], Daojin Li*[b], Xiping Tian[a]

[a] School of Food and Drug, Luoyang Normal University, Luoyang, 471934, P. R.China.

[b] College of Chemistry and Chemical Engineering, and Henan Key Laboratory of Fuction-Oriented Porous Materials, Luoyang Normal University, Luoyang 471934, P. R. China

Supporting Figures

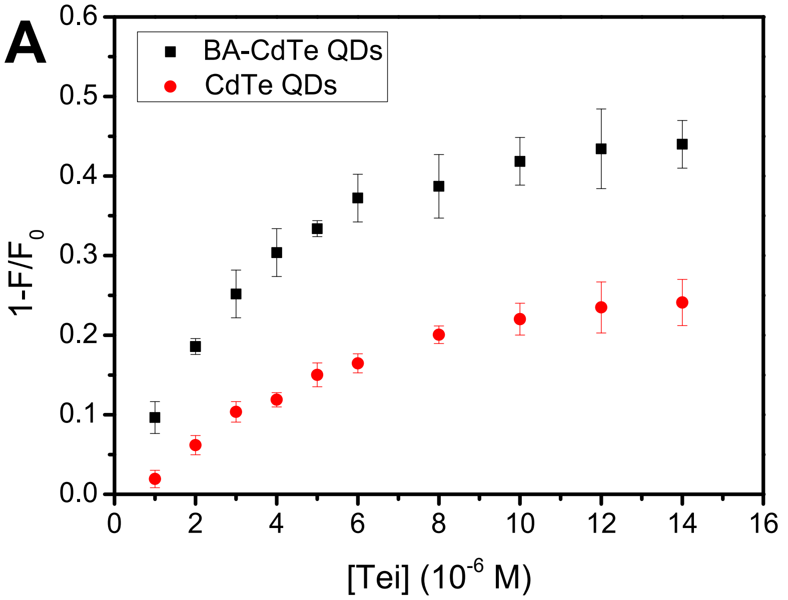

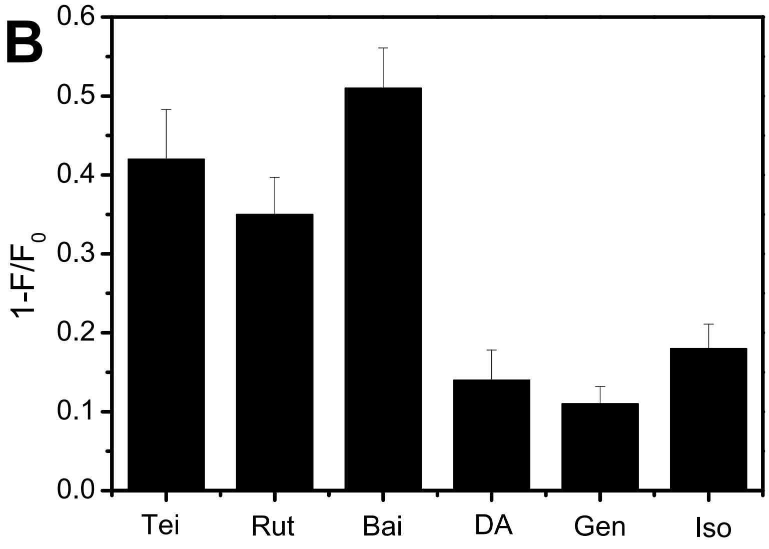

Figure S1 Fluorescence intensity change of CdTe QDs and QDs@APBA with various amount of Tei (A) and binding behaviors of cis-diol-containing compounds (Tei, Rut and Bai) and non-cis-diol-containing compounds (DA, Gen and Iso) on the QDs@APBA (B). [Tei] = [Rut] = [Bai] = [DA] = [Gen] = [Iso] = 1.7×10^{-5} M.

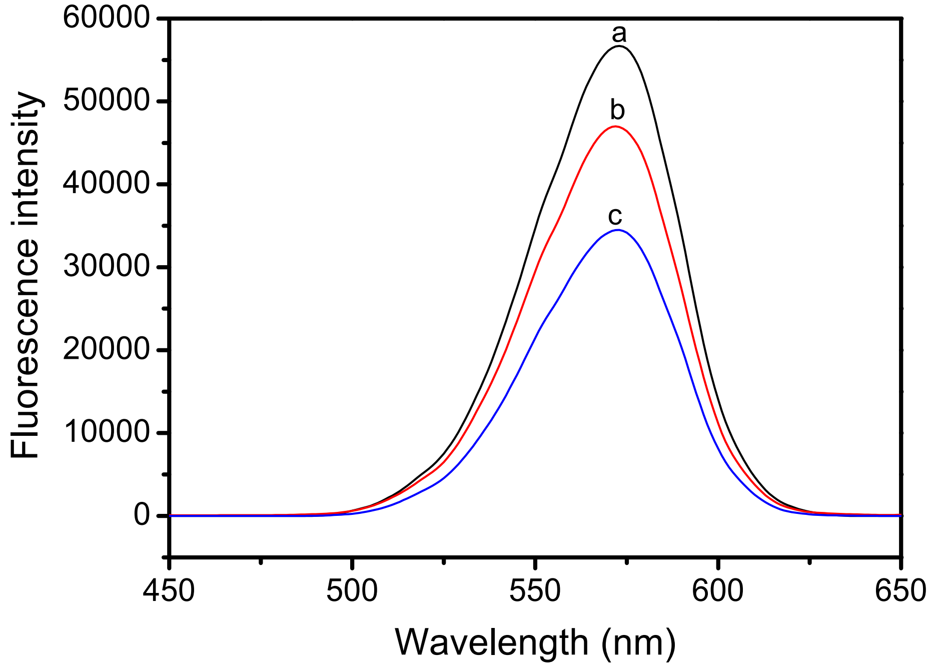

Figure S2 Fluorescence emission spectra of bare CdTe QDs (a), QDs@APBA (b) and QDs@APBA@MIPs (c).

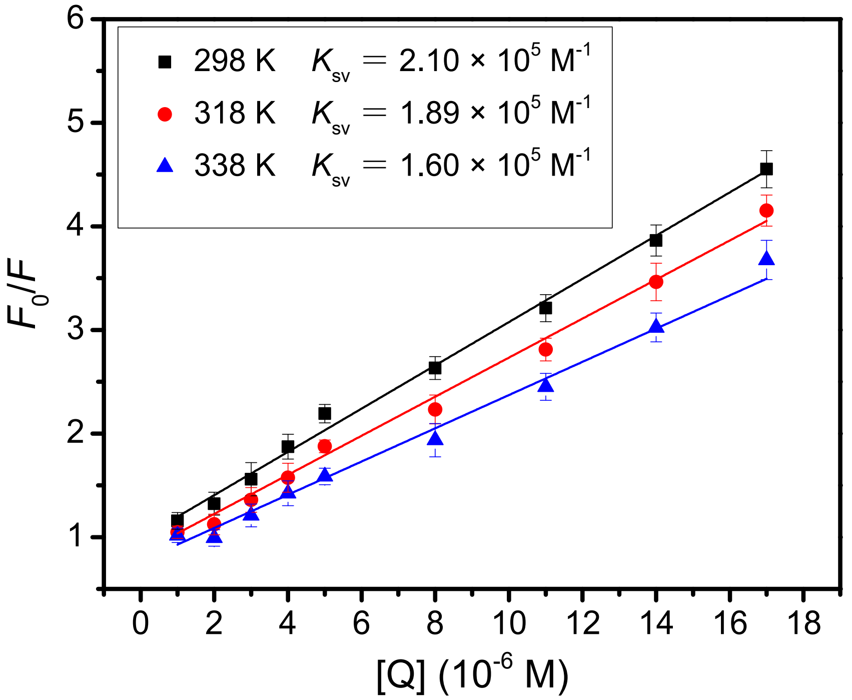

Figure S3 Stern–Volmer plots for fluorescence quenching of QDs@APBA@MIPs towards Tei at different temperatures in pH 7.0.

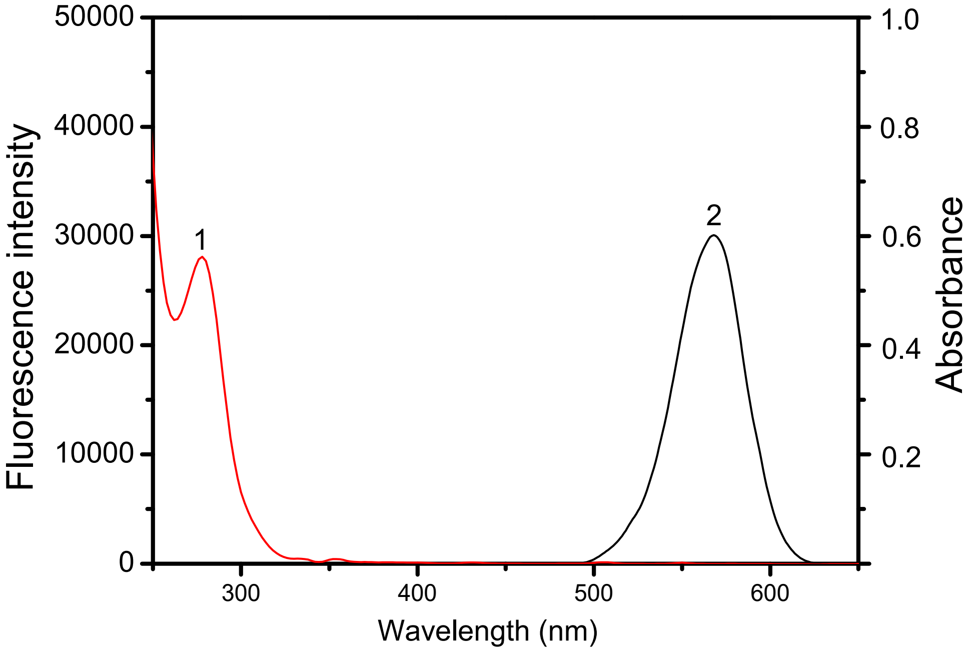

Figure S4 The UV–Vis absorption of Tei (1) and fluorescence emission of QDs@APBA@MIPs (2).

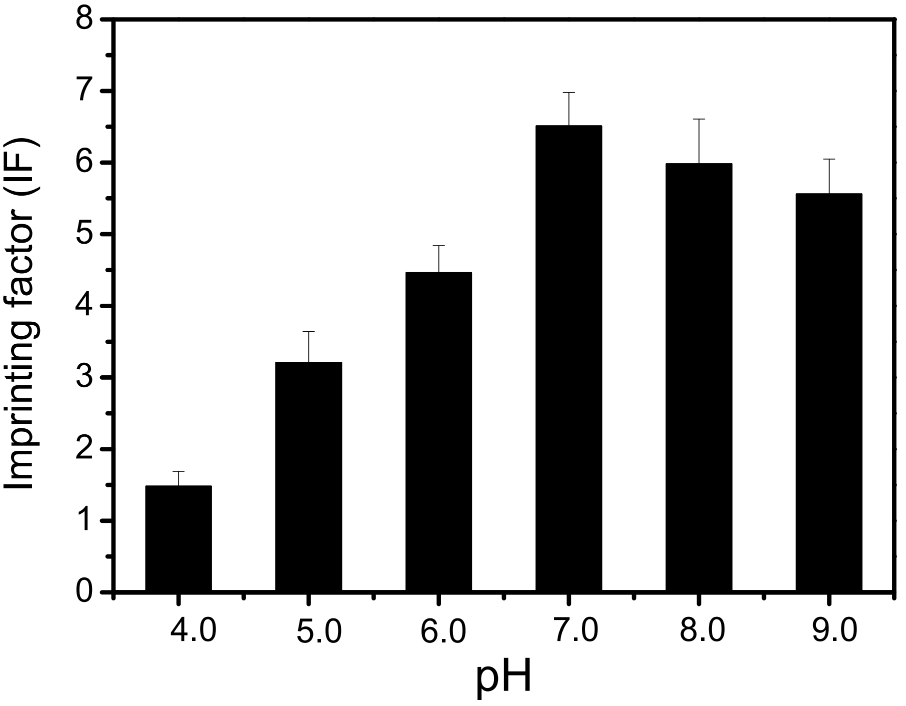

Figure S5 Effect of pH on imprinting factor (IF) of QDs@APBA@MIPs.
